# *SRSF2* mutation cooperates with *ASXL1* truncated alteration to accelerate leukemogenesis

**DOI:** 10.1038/s41375-023-02094-6

**Published:** 2023-11-28

**Authors:** Pinpin Sui, Guo Ge, Shi Chen, Jiaojiao Bai, Ivan P. Rubalcava, Hui Yang, Ying Guo, Peng Zhang, Ying Li, Edward A. Medina, Mingjiang Xu, Omar Abdel-Wahab, Robert Bradley, Feng-Chun Yang

**Affiliations:** 1 Department of Cell Systems & Anatomy, University of Texas Health Science Center at San Antonio, San Antonio, TX USA; 2 Mays Cancer Center, University of Texas Health Science Center at San Antonio, San Antonio, TX USA; 3 Department of Molecular Medicine, University of Texas Health Science Center at San Antonio, San Antonio, TX USA; 4 Department of Pathology and Laboratory Medicine, University of Texas Health Science Center at San Antonio, San Antonio, TX USA; 5 Human Oncology and Pathogenesis Program, Memorial Sloan Kettering Cancer Center, New York, NY USA; 6 Computational Biology Program, Public Health Sciences Division and Basic Sciences Division, Fred Hutchinson Cancer Center, Seattle, WA USA

**Keywords:** Acute myeloid leukaemia, Cancer models, Haematopoietic stem cells, Cancer genomics

**To the Editor:**

*Additional sex combs-like 1 (ASXL1)* gene is highly mutated in a spectrum of myeloid malignancies, including ~49% of chronic myelomonocytic leukemia (CMML) [1], ~10% of acute myeloid leukemia (AML) [2], ~21% of myelodysplastic syndromes (MDS) [3], ~10% of myeloproliferative neoplasms (MPN) [4], and ~8% of juvenile myelomonocytic leukemia (JMML) [5]. The majority of *ASXL1-*mutated patients had other concurrent gene mutations, and splicing factors (*SRSF2*, *U2AF1*, *ZRZR2*, *SF3B1*) were most frequently mutated in myeloid malignancies [6, 7]. Of note, patients with a cooccurring mutation of *ASXL1* and splicing factor mutations have a worse prognosis than patients with either mutation alone or without both mutations [8], suggesting a possible synergistic effect of the two mutations in myeloid malignancy progression.

**Fig. 1:**
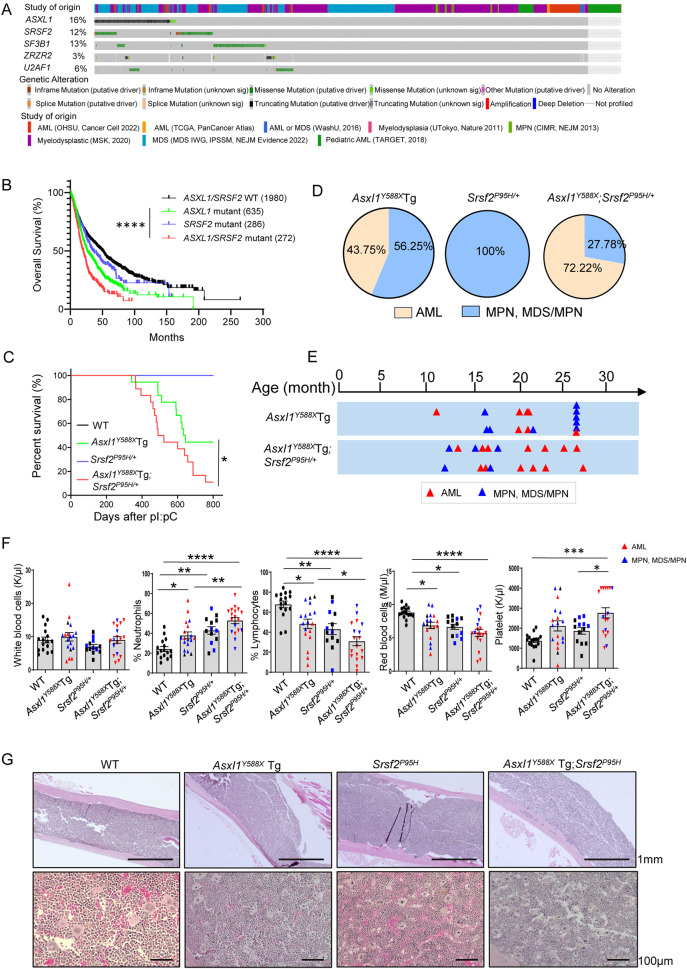
*Srsf2*^*P95H/+*^ mutation exacerbates *Asxl1*^*Y588X*^Tg-induced leukemogenesis. **A** Mutual exclusivity analysis of *ASXL1* mutation and splicing factors mutation (*SRSF2*, *SF3B1*, *U2AF1* and *ZRSR2*). All 10 377 samples with myeloid malignancies collected in cBioPortal were used. **B** Overall survival analysis for 3 323 treatment-naive MDS samples, which were divided into four genotypes (Kaplan–Meier curves with log-rank test). **C** Survival analysis for the mice with different genotypes (Kaplan–Meier curves with log-rank test). The follow-up time is 800 days from the final pIpC injection. **D** For each genotype, the distribution of disease types (leukemia or MPN, MDS/MPN) in all diseased mice. **E** Timeline of disease progression in diseased *Asxl1*^*Y588X*^Tg mice and *Asxl1*^*Y588X*^Tg;*Srsf2*^*P95H/+*^ mice. The red and blue triangle indicates that the onset type is AML and MPN, MDS/MPN, respectively. **F** PB counts showing the numbers of WBCs, neutrophils, lymphocytes, red blood cells, and platelets in WT, *Asxl1*^*Y588X*^Tg, *Srsf2*^*P95H/+*^ and *Asxl1*^*Y588X*^Tg;*Srsf2*^*P95H/+*^ mice. **G** Representative H&E stained femur sections are shown. Scale bar, 1 mm (top); 100 μm (bottom). *P < 0.05; **P < 0.01; ***P < 0.001; ****P < 0.0001.

To assess the impact of concomitant alterations of *ASXL1* and splicing factors in accelerating the progression and aggressiveness of myeloid malignant, we performed mutual exclusivity analysis using 10 377 myeloid malignancies (https://www.cbioportal.org/) for *ASXL1* and splicing factors mutations. We found significant mutation co-occurrence between *ASXL1* and *SRSF2*, *U2AF1*, or *ZRSR2* (log2 Odds Ratio: 1.974, 1.755, 1.177, respectively) (Fig. 1A, Supplementary Table S1). *SRSF2* is most frequently co-mutated with *ASXL1* (*SRSF2* mutation in 28.07% *ASXL1*-mutated patients). In addition, patients with both *ASXL1* and *SRSF2* mutations had unique genetic characteristics and worse survival than patients with *ASXL1* mutation only, *SRSF2* mutation only, and neither (3 323 treatment-naive MDS samples [9], Supplementary Table S2, Fig. 1B, Supplementary Fig. 1A).

*ASXL1* is mainly mutated in the last exon in the form of nonsense or frameshift, resulting in C-terminally truncated mutant proteins, and its mutations are always associated with aggressive disease and poor prognosis [1, 3]. To further decipher the impact of *SRSF2* mutation on disease progression in *ASXL1*-mutated malignancies, we next crossed the *Asxl1*^*Y588X*^Tg [10] with *Mx1Cre*^+^;*Srsf2*^*P95H/+*^ mice [11] to generate *Asxl1*^*Y588X*^Tg;*Mx1Cre*^+^;*Srsf2*^P95H/+^ mice. The mutation of *Srsf2* (*Srsf2*^*P95H/+*^*)* was induced by polyinosine-polycytidine (pIpC) injection (Supplementary Fig. 1B-D) [10, 11]. *Asxl1*^*Y588X*^Tg;*Srsf2*^*P95H/+*^ mice had a significantly shorter survival rate and a higher rate of myeloid leukemogenesis (72.22%) compared to *Asxl1*^*Y588X*^Tg, *Srsf2*^*P95H/+*^ and WT mice (Fig. 1C–E). The AML onset time of *Asxl1*^*Y588X*^Tg;*Srsf2*^*P95H/+*^ mice was 21.3 (13.1–27.2) months with a blast percentage of 43.65% (23.50–58.82%) in bone marrow (BM) (Supplementary Fig. 1E). While the peripheral blood (PB) counts revealed a comparable overall number of white blood cells among the four genotypes of mice, *Asxl1*^*Y588X*^Tg;*Srsf2*^*P95H/+*^ mice had higher neutrophil and platelet counts and lower lymphocyte and red blood cell counts compared to other genotypes of mice (Fig. 1F, Supplementary Fig. 1F). Histologic analysis of the femur, spleen, and liver sections of *Asxl1*^*Y588X*^Tg;*Srsf2*^*P95H/+*^ mice demonstrated pronounced blast cells and myeloid cell infiltration (Fig. 1G, Supplementary Fig. 1G). Analysis of BM cytospin preparations also revealed increased blast cells in *Asxl1*^*Y588X*^Tg;*Srsf2*^*P95H/+*^ mice compared to other genotypes of mice (Supplementary Fig. 2A). Together, these data demonstrated that *Srsf2*^*P95H/+*^ mutation exacerbates *Asxl1*^*Y588X*^Tg-induced leukemogenesis.

**Fig. 2:**
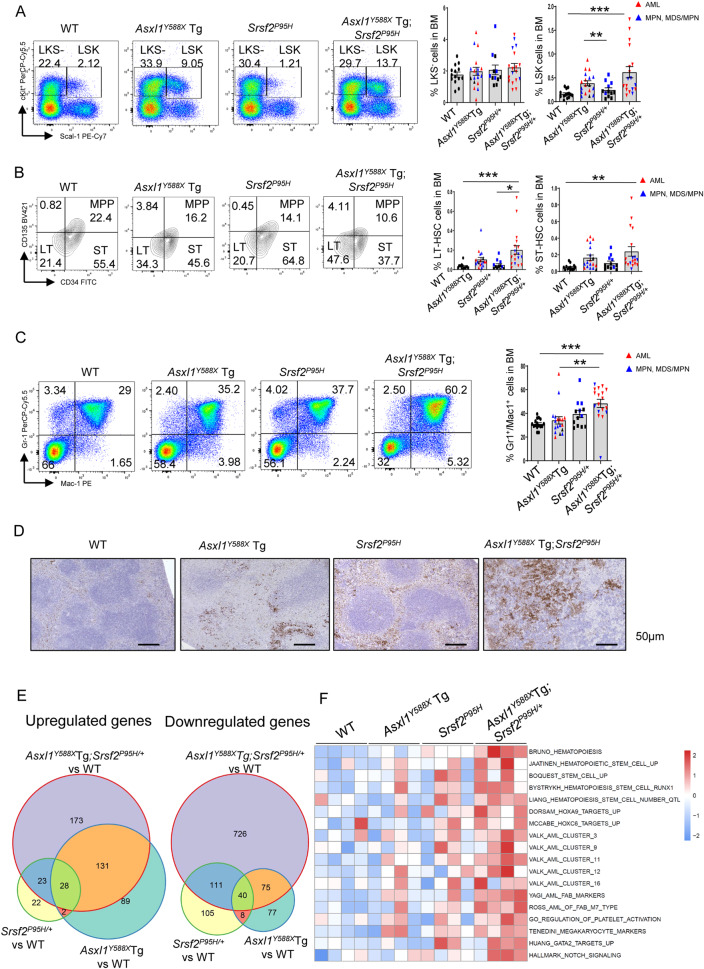
Co-existence of *Srsf2*^*P95H/+*^ and *Asxl1*^*Y588X*^Tg mutation alters the function of HSC/HPCs. **A** Flow cytometric analysis of HSC/HPCs in BM cells from representative mice of each genotype and quantification of the percentages of LSK and LKS^-^ cells. **B** Flow cytometric analysis of LSK cells in BM cells from representative mice of each genotype and quantification of the percentage of LT-HSC and ST-HSC. **C** Flow cytometric analysis of myeloid cells in BM cells from representative mice of each genotype and quantification of the percentage of Gr1^+^/Mac1^+^ cells. **D** Representative MPO staining of spleen sections is shown. Scale bar, 50 μm. **E** The overlap of DEGs of *Asxl1*^*Y588X*^Tg, *Srsf2*^*P95H/+*^ and *Asxl1*^*Y588X*^Tg;*Srsf2*^*P95H/+*^ mice. **F** GSVA score distribution of representative pathways among all four genotypes (scaled among all 16 samples). *P < 0.05; **P < 0.01; ***P < 0.001.

The dysfunctional behavior of hematopoietic stem/progenitor cells (HSC/HPCs) stands as a principal factor in leukemogenesis. Flow cytometric analyses revealed increased frequencies of Lin^−^Sca1^+^cKit^+^ (LSK) cells and long-term (LT)-HSC in the BM of *Asxl1*^*Y588X*^Tg;*Srsf2*^*P95H/+*^ mice compared to other groups of mice (Fig. 2A, B). Furthermore, the frequency of the myeloid population (Gr1^+^/Mac1^+^) was significantly increased in the BM of *Asxl1*^*Y588X*^Tg;*Srsf2*^*P95H/+*^ compared with *Asxl1*^*Y588X*^Tg and WT mice (Fig. 2C). MPO staining of spleen sections confirmed myeloid cell enrichment in *Asxl1*^*Y588X*^Tg;*Srsf2*^*P95H/+*^ mice (Fig. 2D). In contrast, significantly decreased frequencies of CD71^+^/Ter119^+^ erythroid cells in the BM, CD4^+^ cells, CD8^+^ cells, and B220^+^ cells in the spleen were found in *Asxl1*^*Y588X*^Tg;*Srsf2*^*P95H/+*^ mice (Supplementary Fig. 3A–E). These results indicate that *Srsf2* mutation in *Asxl1*^*Y588X*^Tg mice increases the HSC pool and promotes more severe biased myeloid commitment. To further identify the mechanisms of *Asxl1*^*Y588X*^Tg;*Srsf2*^*P95H/+*^-induced leukemogenesis, we carried out RNA-sequencing on sorted LSK from WT, *Asxl1*^*Y588X*^Tg, *Srsf2*^*P95H/+*^ and *Asxl1*^*Y588X*^Tg;*Srsf2*^*P95H/+*^ BM cells (*n* = 4 for each genotype, five months after pIpC injection). A significant difference in the transcriptome profile was observed amongst *Asxl1*^*Y588X*^Tg;*Srsf2*^*P95H/+*^ mice and *Asxl1*^*Y588X*^Tg, *Srsf2*^*P95H/+*^ LSK cells (Supplementary Fig. 4A), although several AML-associated pathways, such as HOXA9/MEIS1 targets and MYC pathway, were significantly upregulated in all three genotypes compared with WT mice (Supplementary Fig. 4B). 339, 450, and 1 307 differentially expressed genes (DEGs) were identified in *Asxl1*^*Y588X*^Tg, *Srsf2*^*P95H/+*^, and *Asxl1*^*Y588X*^Tg;*Srsf2*^*P95H/+*^ mice, respectively (|fold change | > 2 & FDR < 0.05). Although most DEGs of *Asxl1*^*Y588X*^Tg and *Srsf2*^*P95H/+*^ mice were found in *Asxl1*^*Y588X*^Tg;*Srsf2*^*P95H/+*^ mice, 48.75% up-regulated genes and 76.26% down-regulated genes in *Asxl1*^*Y588X*^Tg;*Srsf2*^*P95H/+*^ cells were specifically identified such as *Meis2*, *Sox18*, and *Id3* (Fig. 2E, Supplementary Fig. 4C, D). Scoring the pathways among all samples revealed a specific upregulation of HSC, AML, and megakaryocyte-related pathways in *Asxl1*^*Y588X*^Tg;*Srsf2*^*P95H/+*^ LSK cells (Fig. 2F). Regardless of *SRSF2* being an important splicing factor, we did not identify significantly differential splicing abnormalities in *Asxl1*^*Y588X*^Tg;*Srsf2*^*P95H/+*^ and *Srsf2*^*P95H/+*^ cells (Supplementary Fig. 4E). These data suggested that the co-existence of SRSF2 ^P95H^ and ASXL1 ^aa1-587^ induced a malignant signature, which leads to the dysregulation of HSC/HPCs.

In summary, this study demonstrated that co-occurring mutations of *Asxl1* and *Srsf2* accelerate the development and enhance the severity of myeloid malignancies. Although the proportion of monocytes in the PB of *Asxl1*^*Y588X*^Tg;*Srsf2*^*P95H/+*^ mice is not significantly distinct from *Asxl1*^*Y588X*^Tg and *Srsf2*^*P95H/+*^, it is significantly higher than that of WT, which is consistent with the report of monocytic differentiation in *ASXL1* and *SRSF2* double-mutated AMLs by Johnson et al. [12]. Mechanistically, the *Asxl1*^*Y588X*^Tg;*Srsf2*^*P95H/+*^ induces an increase in the HSC/HPC pool and a biased commitment to myeloid lineage, along with upregulated HSC and AML-associated malignant signature in double mutated mice. Future studies of the contribution of alternative splicing to leukemogenesis in aged *Asxl1*^*Y588X*^Tg;*Srsf2*^*P95H/+*^ mice are warranted.

### Supplementary information

Supplementary file
