# Supplementary material for: SRSF2 mutation cooperates with ASXL1 truncated alteration to accelerate leukemogenesis
Source: Leukemia. 2023 Nov 28;38(2):408–11. doi: 10.1038/s41375-023-02094-6 (PMC10844088; doi:10.1038/s41375-023-02094-6)

Supplementary Materials and methods

Mouse models and Study approval. The generation of *Asxl1*^{Y588X}Tg and *Srsf2*$^{P95H/+}$ has been previously described (1, 2). All of the mice were on a C57BL/6 background. *Mx1Cre*–induced gene deletion was done by intraperitoneal injection of pIpC (10 mg/kg, InvivoGen, San Diego, CA) three times every other day at the age of 1 month for all mice. The genotyping PCR primers are listed in Supplemental Table S4. All animal experiments were conducted in accordance with the National Institutes of Health's guidelines on animal care and use. Experiment protocols were approved by the Institutional Animal Care and Use Committee of UT Health San Antonio. All animals received humane care in compliance with the National Institutes of Health Guide for the Care and Use of Laboratory Animals.

Phenotypic analyses of the hematopoiesis in mice. PB was collected by retro-orbital bleeding and subjected to automated blood count (Element HT5, Heska, Loveland, CO). May-Grünwald-Giemsa stained PB smears and cytospins of bone marrow or spleen cells were used for morphological and lineage differential analyses. Hematoxylin and eosin (H&E) and Myeloperoxidase (MPO) stained tissue sections (femur, spleen, and liver) were used for histopathology analyses, and all slides were visualized under a Keyence BZ-X810 microscope (1).

Flow cytometry and cell sorting. Total white blood cells were obtained after lysis of PB with red cell lysis buffer (Qiagen, 158904). Single-cell suspensions from PB, BM and spleen were stained with panels of fluorochrome-conjugated antibodies (Supplemental Table S5). All flow cytometric analyses were performed using a BD FACS Celesta flow cytometer (BD Biosciences) and all data were analyzed by FlowJo_V10 software (1).

Western blot assay. The whole cell lysates were prepared using the RIPA buffer (MilliporeSigma, St. Louis, MO) and then resolved on NuPAGE 4-12% Bis-Tris Gels (Invitrogen, Carlsbad, CA).

Immunoblotting was performed with the antibody of FLAG, β-actin and SRSF2. Signals were detected using the Prometheus ProSignal ECL reagents (Genesee Scientific, San Diego, CA). Images were taken on a ChemiDoc MP Imaging System (Bio-Rad Laboratories, Hercules, CA).

RNA-Seq and data analysis. BM LSK cells from WT, *Asxl1*^{Y588X}Tg, *Srsf2*$^{P95H/+}$ and *Asxl1*^{Y588X}Tg;*Srsf2*$^{P95H/+}$ were purified (n = 4 for each genotype, 5 months after pIpC injection). Total RNA from individual mice was isolated with RNeasy Plus Mini Kit (Qiagen) and subjected to RNA library preparation following a non-stranded protocol with the NEB Ultra RNA kit. All libraries were sequenced at a read length of pair-end 100bp with final reads over 85 million per sample. The raw data from all 16 samples was trimmed by Trimmomatic (v 0.38)(3) to remove adapter and low-quality reads, and remained clean reads were aligned to the mouse genome (mm10) separately using STAR (v 2.7.0e)(4). The raw read counts of each gene were calculated by HTSeq (v 0.11.2)(5) and converted to transcripts per million (TPM) format. Then, the count matrix was used to identify differentially expressed genes by DESeq2 (6) with a cutoff of false discovery rate (FDR) < 0.05 and |fold change| > 2. Gene Set Enrichment Analysis (GSEA)(7) was performed to identify differential expressed pathways among different genotypes and Gene Set Variation Analysis (GSVA) was performed to score the pathways' activity among all 16 samples using R package GSVA(8). Alternative splicing analysis was performed to identify differential alternative splicing events among different genotypes using rMATS(9) with a cutoff of FDR < 0.05 and |IncLevelDifference| > 0.1.

Quantitative PCR analysis. Total RNA was purified with RNeasy Mini Kit (Qiagen) according to the manufacturer's instructions (n=4 for each genotype). A total of 500 ng RNA was subjected to reverse-transcription using High Capacity cDNA Reverse Transcription Kits (Applied Biosystems) and qPCR was performed using an Applied Biosystems QuantStudio 3 system with the Fast SYBR Green Master Mix (Applied Biosystems). qPCR amplifications were performed in triplicate

for *Meis2*, *Sox18* and *Id3* along with parallel measurements of β-actin cDNA (internal control). All primer sequences are listed in Supplementary Table S4.

Statistical analysis. Statistical significance was determined by one-way ANOVA for comparisons of all four groups of mice, or two-tailed unpaired t-test for comparisons between two groups of mice. Log-rank test was used for the assessment of survival difference. P values of less than 0.05 are considered significantly different. Statistical analyses were conducted and analyzed using GraphPad Prism 9.0 (GraphPad Software). For phenotype analysis, more than 10 mice per genotype were included. For survival analysis, 18 mice per genotype were included.

Data availability. The RNA-seq data in this study are deposited in Gene Expression Omnibus (GEO) archive under accession of GSE240105.

Reference

1. Yang H, Kurtenbach S, Guo Y, Lohse I, Durante MA, Li J, et al. Gain of function of ASXL1 truncating protein in the pathogenesis of myeloid malignancies. Blood. 2018;131(3):328-41.
2. Kim E, Ilagan JO, Liang Y, Daubner GM, Lee SC, Ramakrishnan A, et al. SRSF2 Mutations Contribute to Myelodysplasia by Mutant-Specific Effects on Exon Recognition. Cancer Cell. 2015;27(5):617-30.
3. Bolger AM, Lohse M, Usadel B. Trimmomatic: a flexible trimmer for Illumina sequence data. Bioinformatics. 2014;30(15):2114-20.
4. Dobin A, Davis CA, Schlesinger F, Drenkow J, Zaleski C, Jha S, et al. STAR: ultrafast universal RNA-seq aligner. Bioinformatics. 2013;29(1):15-21.
5. Anders S, Pyl PT, Huber W. HTSeq--a Python framework to work with high-throughput sequencing data. Bioinformatics. 2015;31(2):166-9.
6. Love MI, Huber W, Anders S. Moderated estimation of fold change and dispersion for RNA-seq data with DESeq2. Genome Biol. 2014;15(12):550.
7. Subramanian A, Tamayo P, Mootha VK, Mukherjee S, Ebert BL, Gillette MA, et al. Gene set enrichment analysis: A knowledge-based approach for interpreting genome-wide expression profiles. 2005;102(43):15545-50.
8. Hanzelmann S, Castelo R, Guinney J. GSVA: gene set variation analysis for microarray and RNA-seq data. BMC Bioinformatics. 2013;14:7.
9. Shen S, Park JW, Lu ZX, Lin L, Henry MD, Wu YN, et al. rMATS: robust and flexible detection of differential alternative splicing from replicate RNA-Seq data. Proc Natl Acad Sci U S A. 2014;111(51):E5593-601.

10. Bernard E, Tuechler H, Greenberg PL, Hasserjian RP, Arango Ossa JE, Nannya Y, et al. Molecular International Prognostic Scoring System for Myelodysplastic Syndromes. NEJM Evidence. 2022;1(7).

Supplementary Table S1 Mutual exclusivity among ASXL1 and splicing factors within10037 samples with myeloid diseases in cBioPortal.

A	B	Neither	A Not B	B Not A	Both	Log2 Odds Ratio	p-Value	q-Value	Tendency
ASXL1	SRSF2	6261	1025	622	400	1.974	<0.001	<0.001	Co-occurrence
ASXL1	U2AF1	6562	1223	321	202	1.755	<0.001	<0.001	Co-occurrence
ASXL1	ZRSR2	6617	1315	207	93	1.177	<0.001	<0.001	Co-occurrence
ASXL1	SF3B1	5901	1250	982	175	-0.249	0.048	0.054	Mutual exclusivity

Supplementary Table S2 Mutational rates of common mutated genes amongst MDS patients with four different genotypes (3 323 treatment-naive MDS samples(10)).

Genes	*ASXL1/SRSF2* WT	*ASXL1* mutant	*SRSF2*-mutant	*ASXL1/SRSF2* mutant
TET2	25.61%	31.02%	61.74%	45.71%
SF3B1	28.98%	18.22%	5.70%	4.29%
DNMT3A	20.47%	10.84%	11.74%	2.50%
RUNX1	6.34%	23.04%	24.50%	40.36%
TP53	16.43%	5.42%	4.70%	1.43%
STAG2	3.65%	14.16%	9.06%	41.07%
U2AF1	7.26%	19.88%	1.01%	1.07%
EZH2	3.32%	22.59%	0.67%	7.50%
KMT2D	6.97%	6.63%	5.37%	6.79%
BCOR	5.86%	6.48%	9.40%	9.64%
ZRSR2	5.62%	10.39%	3.02%	2.14%
CBL	3.70%	9.34%	11.41%	9.64%
CUX1	3.65%	7.38%	15.10%	8.57%
NF1	4.08%	8.13%	6.71%	10.36%
SETBP1	3.32%	10.09%	5.70%	12.50%
IDH2	2.64%	5.12%	11.74%	20.00%
NRAS	3.60%	6.78%	6.71%	13.93%
KMT2C	5.24%	4.52%	4.03%	5.36%

Supplementary Table S3. Summary of diseased/moribund mice with different genotypes.

Group	Survival (days)	Frequency	BM blasts	Diagnosis and Subclassification
$Asxl1^{Y588X}$Tg	489-800	56.25% (9/16)	<20%	MPN, MDS/MPN
	341-800	43.75% (7/16)	>20%	Myeloid leukemia
$Srsf2^{P95H/+}$	800	100% (5/5)	<20%	MPN, MDS/MPN
$Asxl1^{Y588X}$Tg;$Srsf2^{P95H/+}$	365-523	27.78% (5/18)	<20%	MPN, MDS/MPN
	394-800	72.22% (13/18)	>20%	Myeloid leukemia

Supplementary Table S4. List of primers used in this study.

Name	Forward	Reverse
Asxl1^{Y588X}	ACCCGTCAACGGGACGGAC	CGATCCGGGGGCATATCTGTC
Srsf2-flox	CAACACGGCCGATATCATAA	TACAGTCCTCGTGGGTAGGG
Mx1-Cre	CGGTCCTGCTACAGTTCTGT	TCTTTGACACGGCCTTGGTA
mMeis2	TTCCAGCATCTCACACACCC	TCACTGCTCGATTTGACTGGT
mSox18	TCTCAATTGCAGCCGGACTC	AGCATCAGACAGCGCAGAAA
mId3	GCCCGAGAGAAGGACTGAAC	CGACACCCCATTCTCGGAAA
mActb	GGCTGTATTCCCCTCCATCG	CCAGTTGGTAACAATGCCATGT

Supplementary Table S5. List of antibodies used in this study.

Reagent or Resource	Source	Identifier
Mouse monoclonal anti-FLAG M2	MilliporeSigma	Cat# F3165; RRID: AB_259529
Rabbit polyclonal anti-SRSF2	ThermoFisher	Cat# PA5-12402; RRID: AB_2184941
Mouse monoclonal anti-β-Actin	MilliporeSigma	Cat# A2228; RRID: AB_476697
Mouse lineage antibody cocktail APC	BD Pharmingen	Cat# 558074; RRID: AB_1645213
Rat monoclonal anti-mouse CD117 (cKit) PerCP-Cy5.5	BD Pharmingen	Cat# 560557; RRID: AB_1645258
Rat monoclonal anti-mouse Ly-6A/E (Sca1) PE-Cy7	BD Pharmingen	Cat# 558162; RRID: AB_647253
Rat monoclonal anti-mouse CD34 FITC	BD Pharmingen	Cat# 553733; RRID: AB_395017
Rat monoclonal anti-mouse CD16/32 APC-Cy7	BioLegend	Cat# 101327; RRID: AB_1967102
Rat monoclonal anti-mouse CD71 PE	BD Pharmingen	Cat# 553267; RRID: AB_394744
Rat anti-mouse TER-119 APC	BD Pharmingen	Cat# 557909; RRID: AB_398635
Rat monoclonal anti-mouse Ly-6G and Ly-6C (Gr1) PerCP-Cy5.5	BD Pharmingen	Cat# 550954; RRID: AB_393977
Rat monoclonal anti-mouse CD11b (Mac1) PE	BD Pharmingen	Cat# 553311; RRID: AB_394775
Rat monoclonal anti-mouse CD4 PerCP-Cy5.5	BD Pharmingen	Cat# 550954; RRID: AB_393977
Rat monoclonal anti-mouse CD8a PE	BD Pharmingen	Cat# 553033; RRID: AB_394571
Rat monoclonal anti-mouse CD45R/B220 APC	BD Pharmingen	Cat# 553092; RRID: AB_398531
Mouse monoclonal anti-Myeloperoxidase/MPO	R&D systems	Cat# MAB3174

Supplementary Figure Legends

Figure 1. *Srsf2*$^{P95H/+}$ mutation exacerbates *Asxl1*^{Y588X}Tg-induced leukemogenesis. **A** Leukemia-free survival analysis for 3323 treatment-naive MDS samples, which were divided into four genotypes (Kaplan–Meier curves with log-rank test). **B** Schematic diagram of the generation of *Asxl1*^{Y588X}Tg;*Srsf2*$^{P95H/+}$ mice. **C** Genotyping results of *Mx1Cre*$^{+}$;*Srsf2*$^{+/+}$ (WT), *Mx1Cre*$^{+}$;*Asxl1*^{Y588X}Tg (*Asxl1*^{Y588X}Tg), *Mx1Cre*$^{+}$;*Srsf2*$^{P95H/+}$ (*Srsf2*$^{P95H/+}$), and *Asxl1*^{Y588X}Tg;*Mx1Cre*$^{+}$;*Srsf2*$^{P95H/+}$ (*Asxl1*^{Y588X}Tg; *Srsf2*$^{P95H/+}$) mice. **D** Western blot results show the expression of Flag (ASXL1$^{aa1-587}$) and SRSF2. **E** The blasts percentages of 13 AML mice with the genotype of *Asxl1*^{Y588X}Tg;*Srsf2*$^{P95H/+}$ evaluated by histologic analyses of cytospin preparations from bone marrow of these mice. Data represents the Mean ± SEM. **F** The level of monocyte, Hemoglobin in PB, body weight, BM cellularity and spleen weight in WT, *Asxl1*^{Y588X}Tg, *Srsf2*$^{P95H/+}$ and *Asxl1*^{Y588X}Tg;*Srsf2*$^{P95H/+}$ mice. **G** Representative H&E staining of liver (top, Scale bar, 50 μm) and spleen sections are shown. (bottom, Scale bar, 50 μm). * $P < .05$; ** $P < .01$; *** $P < .001$; **** $P < .0001$.

Figure 2. A May-Giemsa–stained BM cytospins prepared from representative mice of each genotype.

Figure 3. Co-existence of SRSF2^{P95H} and ASXL1$^{aa1-587}$ mutation induces skewed lineage differentiation. **A** Flow cytometric analysis of erythroid cells in BM cells from representative mice of each genotype and quantification of the percentage of CD117^{+}Ter119^{+} cells. **B-C** Flow cytometric analysis of lymphoid cells in BM cells from representative mice of each genotype and quantification of the percentage of CD4^{+} cells, CD8^{+} cells and B220^{+} cells. D-E Flow cytometric analysis of lymphoid cells in spleen cells from representative mice of each genotype and quantification of the percentage of CD4^{+} cells, CD8^{+} cells and B220^{+} cells. * $P < .05$; ** $P < .01$; *** $P < .001$; **** $P < .0001$.

Figure 4. Transcriptome changes in single and double mutated HSC/HPCs. **A** Heatmap depicting significantly dysregulated genes in *Asxl1*^{Y588X}Tg compared with WT controls, *Srsf2*$^{P95H/+}$ compared with WT controls and *Asxl1*^{Y588X}Tg;*Srsf2*$^{P95H/+}$ compared with WT controls (FDR < 0.05 and |fold change| > 2). **B** Gene set enrichment analysis (GSEA) show the enrichment of HESS_TARGETS_OF_HOXA9_AND_MEIS1_UP, HALLMARK_MYC_TARGETS_V1, and HALLMARK_MYC_TARGETS_V2 in *Asxl1*^{Y588X}Tg, *Srsf2*$^{P95H/+}$ and *Asxl1*^{Y588X}Tg;*Srsf2*$^{P95H/+}$ LK cells respectively. **C** The normalized expression (TPM) of *Meis2*, *Sox18* and *Id3* in LSK cells among four genotypes (n=4 for each genotype). Data represents the Mean ± SEM. Two-tailed unpaired t-test was used for the statistical analysis among *Asxl1*^{Y588X}Tg;*Srsf2*$^{P95H/+}$ and WT control. D Relative expression (RT-qPCR) of target genes in LK cells from different genotypes. Data represents the Mean ± SEM (n=4 for each genotype; two-tailed unpaired t-test). E The overlap of differential expressed alternative splicing events of *Asxl1*^{Y588X}Tg, *Srsf2*$^{P95H/+}$ and *Asxl1*^{Y588X}Tg;*Srsf2*$^{P95H/+}$ mice. All five types of alternative splicing were included (SE, RI, MXE, A3SS and A5SS).

Supplementary Fig. 1

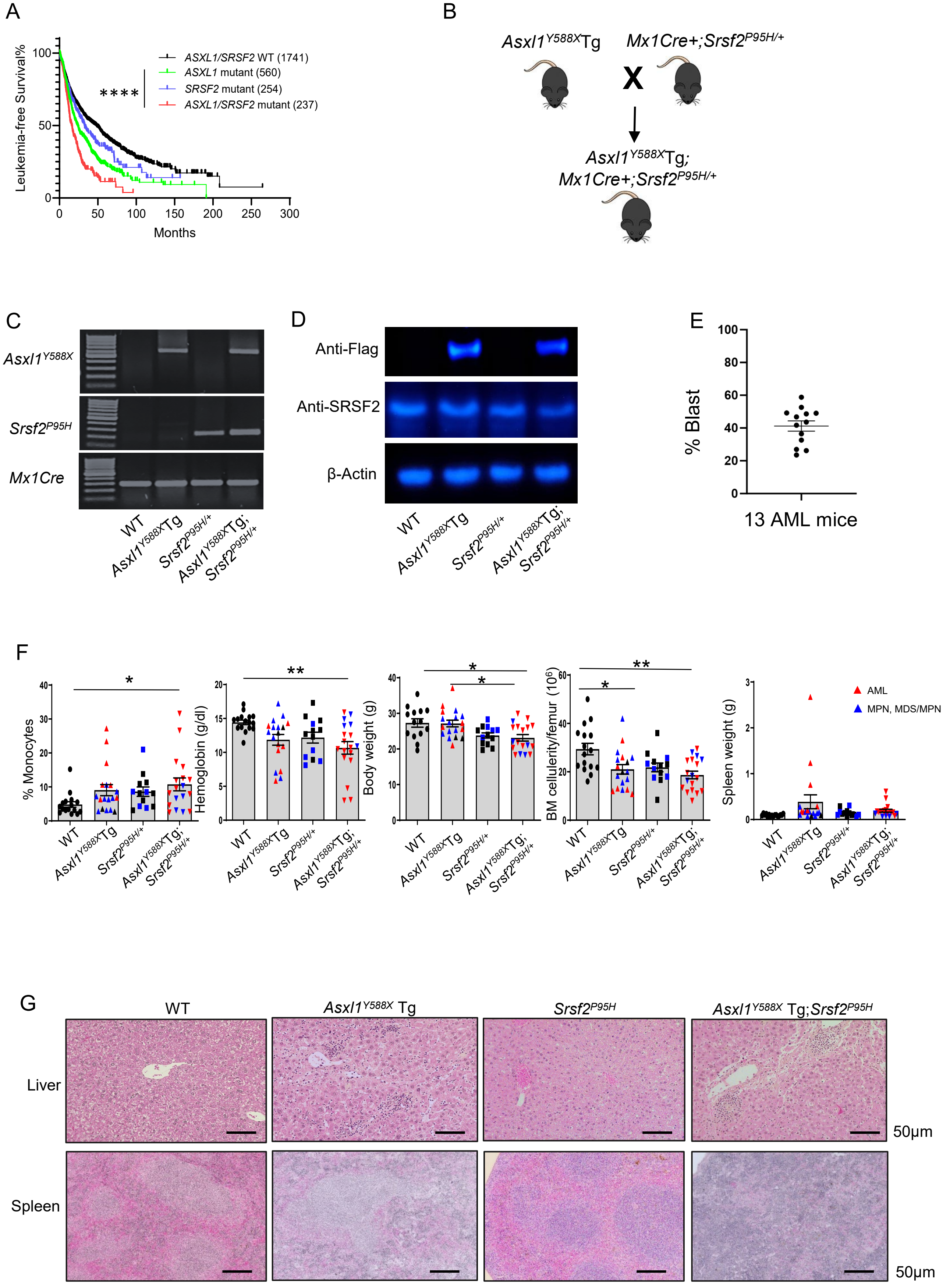

Supplementary Fig. 2

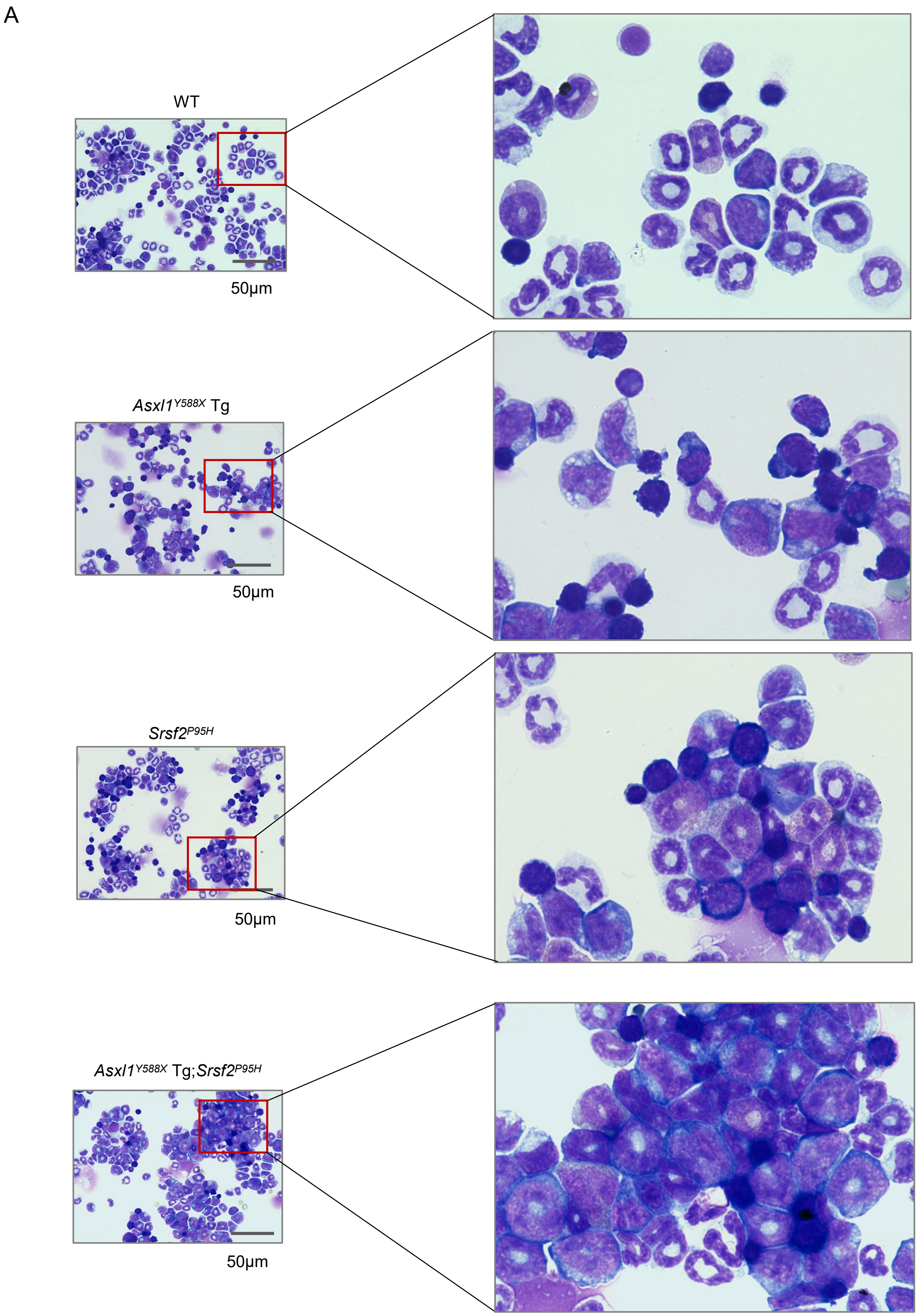

Supplementary Fig. 3

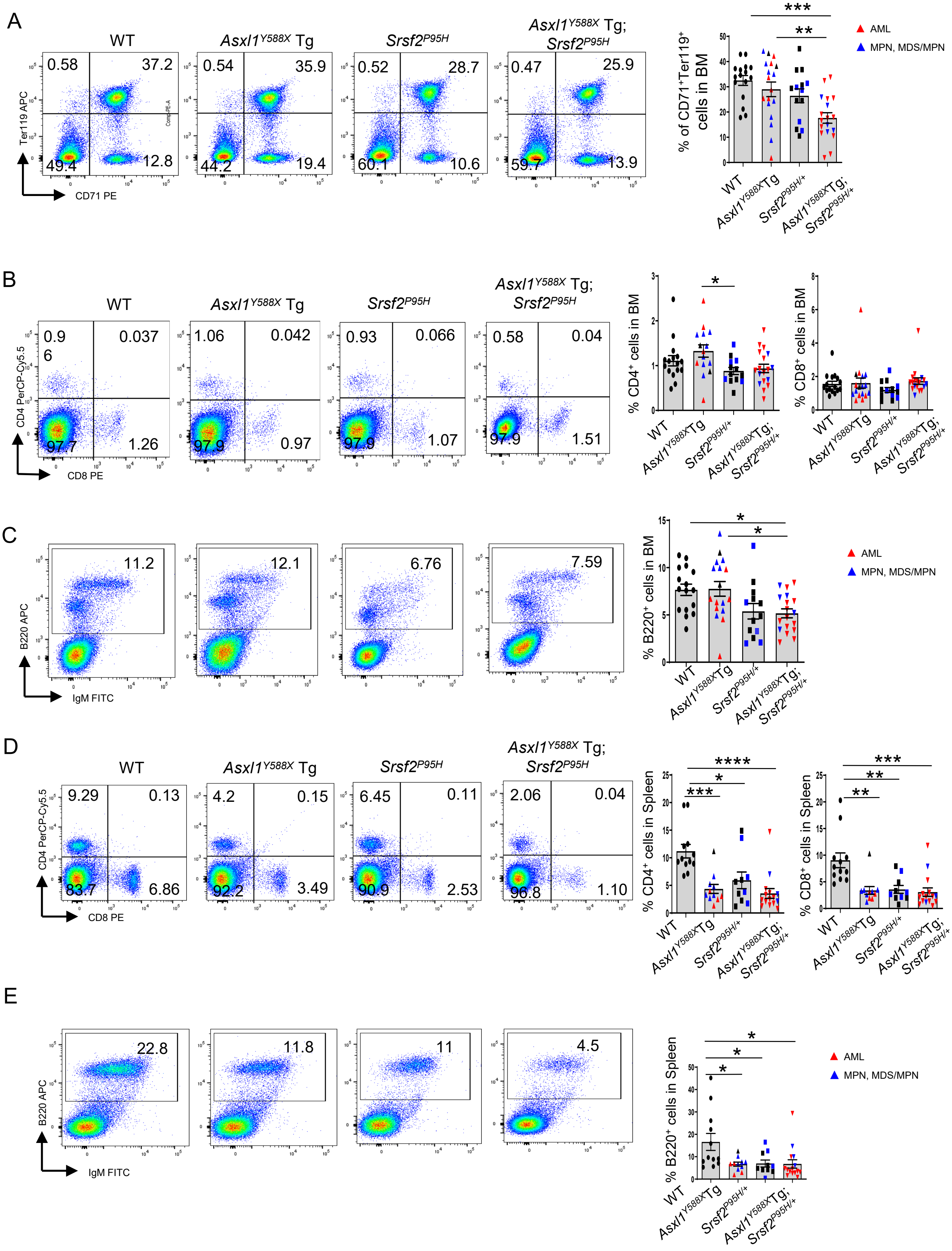

Supplementary Fig. 4

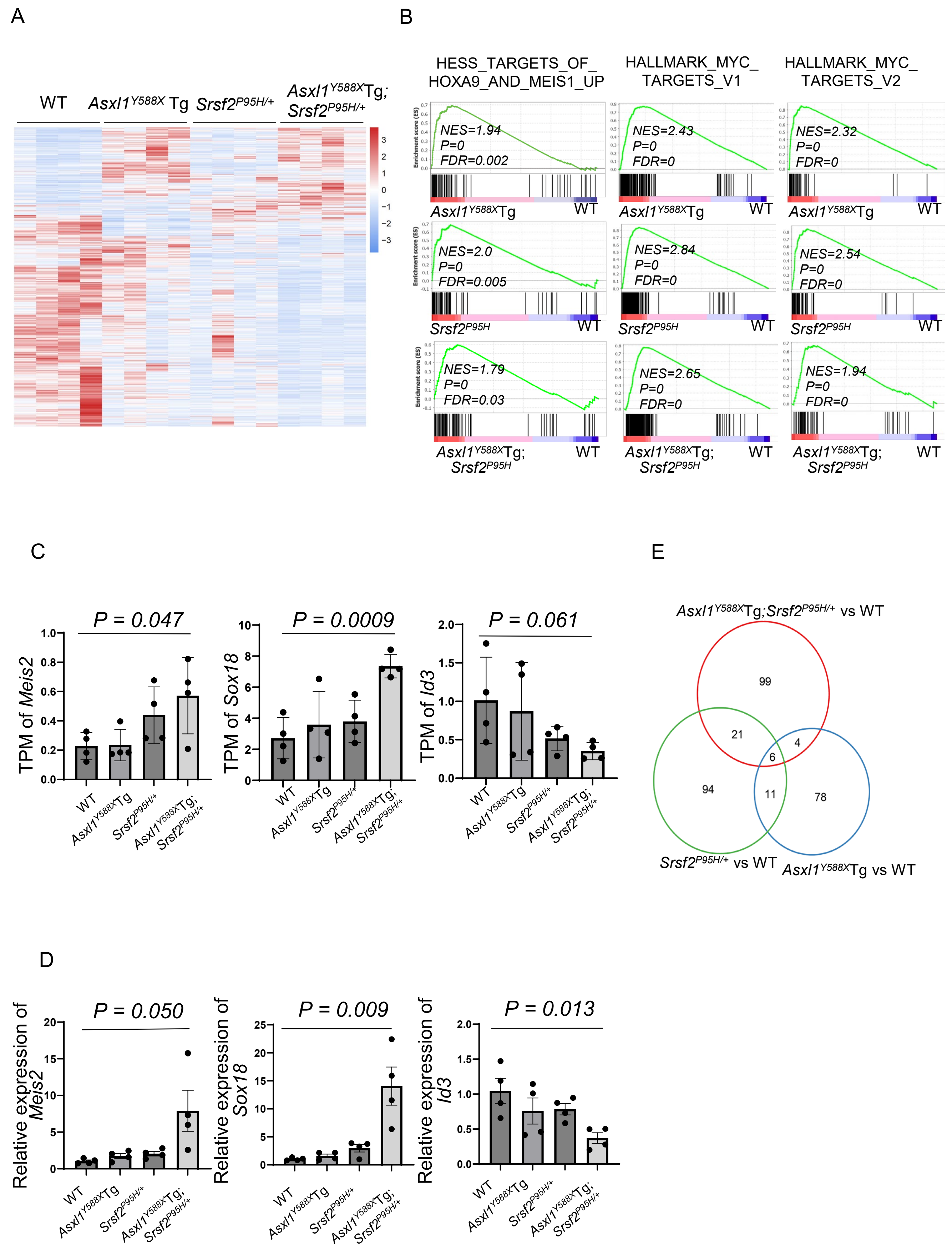

Supplement: Supplementary file 1 — Supplementary file [file 41375_2023_2094_MOESM1_ESM.pdf]
